# A Possible Association of Diindolylmethane with Pulmonary Embolism and Deep Venous Thrombosis

**DOI:** 10.1155/2016/7527098

**Published:** 2016-12-05

**Authors:** Peter V. Bui, Maan Moualla, Dona J. Upson

**Affiliations:** ^1^Department of Internal Medicine, University of New Mexico, Albuquerque, NM, USA; ^2^West Houston Medical Center, Hospital Corporation of America, Houston, TX, USA; ^3^Section of Pulmonary, Critical Care, and Sleep Medicine, Department of Internal Medicine, Raymond G. Murphy VA Medical Center, Albuquerque, NM, USA; ^4^Division of Pulmonary, Critical Care, and Sleep Medicine, Department of Internal Medicine, University of New Mexico, Albuquerque, NM, USA

## Abstract

*Introduction.* 3,3′-Diindolylmethane is available as a supplement in the United States for “cancer prevention” and “augmentation of physical fitness.” A derivative of indole-3-carbinol found in plants, diindolylmethane, binds to receptors associated with the sex steroid pathways and has unclear effects on estrogen and androgen physiology. We present a patient who had been taking diindolylmethane and developed right lower extremity deep venous thrombosis and bilateral pulmonary embolism.* Case Presentation.* A 65-year-old man presented with swelling, erythema, and warmth of his right lower extremity for three to four weeks. He had been taking diindolylmethane one tablet daily for three to four months. Risk factors for venous thromboembolism included tobacco use, personal history of possible pulmonary embolism, body mass index, and age. Imaging studies found extensive deep venous thrombosis in his right lower extremity and bilateral pulmonary embolism with probable right middle lobe infarction. Follow-up imaging showed chronic deep venous thrombosis in his right lower extremity.* Discussion.* As suggested in this single case, patients who take diindolylmethane may be at greater risk for venous thromboembolism. Further reports and studies are necessary in order to elucidate this possible association. Clinicians should question patients about supplements in the setting of venous thromboembolism.

## 1. Introduction

The incidence of first-time venous thromboembolism (VTE) in the United States is 71 to 117 cases per 100,000 persons [1]. Within 10 years of an initial VTE, 17.6% of patients have been found to develop recurrent VTE [2]. Risk factors for VTE include older age, above-normal body mass index, personal or family history of VTE, surgery, hospitalization, malignancy, myeloproliferative disease, trauma, immobility, varicose veins, pregnancy or puerperium, postmenopausal hormone therapy, selective estrogen receptor modulators, oral contraceptives (OCPs), extended travel, genetic factors, antiphospholipid syndrome, and tobacco use [3–7]. Postmenopausal patients taking hormone replacement therapy containing estrogen only or a combination of estrogen and progestin have a two- to fourfold increased risk of VTE [8]. 3,3′-Diindolylmethane (DIM), a derivative of indole-3-carbinol found in plant products, has unclear effects on the estrogen and androgen physiology of humans. Most research has focused on breast, cervical, and prostate cancer and has been performed using cell models or animal models, less commonly human studies [9–13]. The effect, if any, of DIM on the formation of VTE is unknown. We describe the first reported case of deep venous thrombosis (DVT) and pulmonary embolism (PE) in a man with risk factors for VTE taking DIM.

## 2. Case Presentation

A 65-year-old man with a remote history of possible PE and current tobacco use presented with swelling, erythema, and warmth of his right lower extremity for three to four weeks (Table 1). He denied cough, hemoptysis, shortness of breath, chest pain, recent travel, prolonged immobilization, or history of malignancy. Approximately ten years ago he was diagnosed with a PE at an unrecallable hospital and did not receive anticoagulation. The events surrounding this self-reported PE could not be verified.

**Figure 1:**
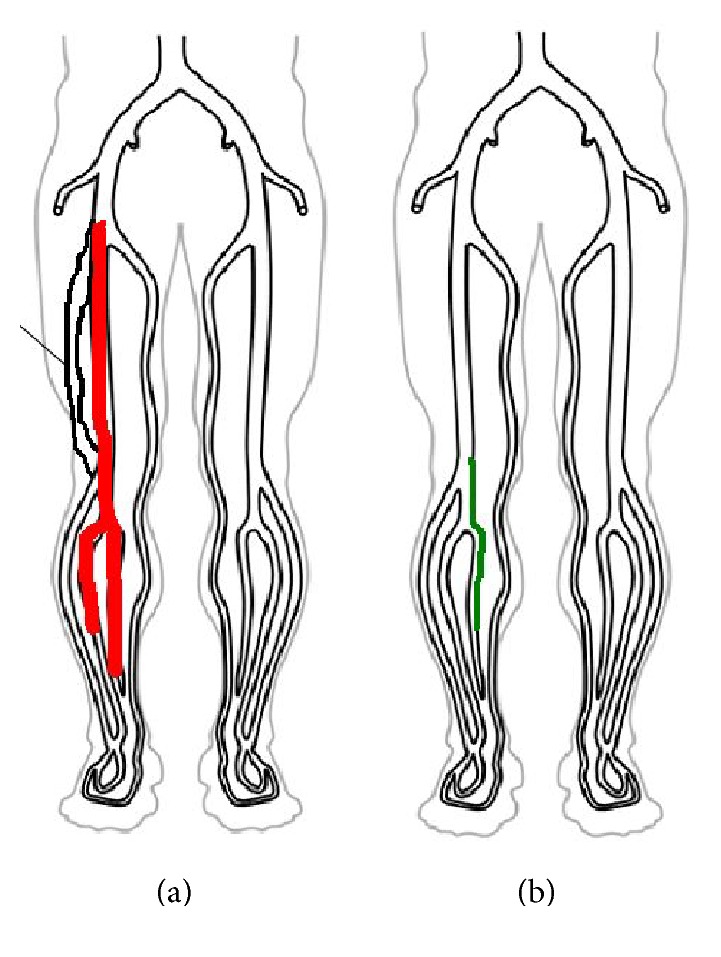
(a) The lower extremity venous Doppler study on the day of admission showed acute deep venous thrombosis in the right common femoral, femoral, popliteal, posterior tibial, and peroneal veins. (b) The lower extremity venous Doppler study five months after discharge showed chronic deep venous thrombosis in the right popliteal and peroneal veins.

Five weeks before presentation, the patient went to the Emergency Department with low back and right buttock pain radiating down to the mid-posterior right calf and was diagnosed with sciatica. Two weeks later, he returned with pleuritic chest pain and exertional dyspnea, thought to be secondary to costochondritis; he left against medical advice. Three weeks later, on the day of admission, he was evaluated by his primary care physician, who obtained a lower extremity venous Doppler study that showed extensive DVT in his right lower extremity (Figure 1(a)).

The patient's medications included bupropion, cholecalciferol, ibuprofen, prazosin, simvastatin, vitamin A, and over-the-counter supplements including DIM. He had used over-the-counter testosterone supplements for six months but discontinued them 13 months before hospitalization. Prior to his hospitalization, he used DIM one tablet daily for three to four months. He did not know the dose, but a common dose is 100 mg per tablet. He had a 60 pack-year smoking history, with cessation for five years prior to resumption one year ago. He had a documented weight loss of 7 kg over one month, but he endorsed a recent increase in exercise, including skiing and weight lifting. Colonoscopy four months earlier found a benign polyp. Prostate specific antigen was less than 3 ng/mL a year before and during hospitalization.

**Figure 2:**
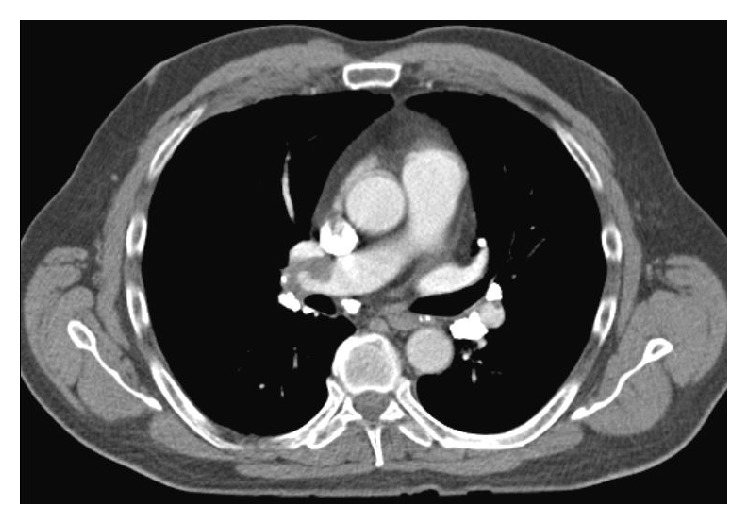
Computed tomography of the chest depicting filling defects in the pulmonary arterial vasculature secondary to pulmonary embolism.

**Figure 3:**
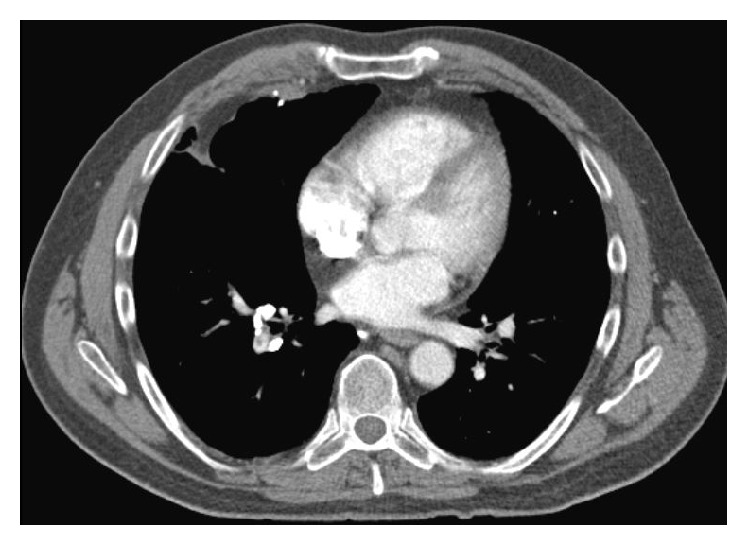
Computed tomography of the chest depicting filling defects in the pulmonary arterial vasculature secondary to pulmonary embolism and probable right middle lobe infarction.

Physical examination revealed a heart rate of 104 beats per minute, blood pressure 123/81 mmHg, respiratory rate 16 breaths per minute, temperature 36.1°C, and oxygen saturation 96% on room air. The right leg was mildly swollen with erythema and warmth. The rest of the examination was unremarkable. Chemistry panel, complete blood count, hepatic function panel, and coagulation studies were normal. PE-protocol computed tomography (CT) of the chest (Figures 2 and 3) showed bilateral pulmonary artery filling defects with marked involvement of the right middle lobe branch with associated probable infarction. He was treated with anticoagulation and counseled on tobacco dependence. Five months later, a lower extremity venous Doppler study found chronic DVT in the right lower extremity (Figure 1(b)).

## 3. Discussion

We describe a patient who developed acute right lower extremity DVT and bilateral PE with right middle lobe infarction and subsequent chronic right lower extremity DVT. Although the time course is unclear, while taking DIM, he may have started developing the DVT five weeks before admission when he presented with right lower extremity complaints and the PE three weeks before admission when he presented with thoracic and respiratory complaints. His risk factors for VTE included older age, an above-normal body mass index, tobacco use, a past history of possible PE, and the unclear estrogenic effects of DIM. With a documented weight loss of 7 kg over one month, he may have had an undiscovered cancer, despite negative age-appropriate cancer screening and no evidence of malignancy on CT angiography. Although causality cannot be demonstrated in this single case, the use of DIM preceded the VTE by several months, and DIM may have contributed to the development of VTE.

DIM is not specifically an estrogen analogue but does bind to the aryl hydrocarbon receptor, which interacts with the estrogen-related pathways, and the estrogen receptor. DIM has both agonist and antagonist properties in estrogen physiology, in addition to having testosterone antagonist properties [9–12, 14–17]. Estrogen analogues modify the milieu of the coagulation factors, anticoagulation system, and fibrinolytic system in a way that promotes VTE, particularly in patients with genetic predispositions [18]. Vandenbroucke et al. found the incidence of venous thrombosis to be 0.8 per 10,000 person-years and 3.0 per 10,000 person-years for non-OCP users and OCP users, respectively, and the relative risk of venous thrombosis to be 3.8 (95% CI 2.4–6.0) for OCP users and even higher for patients with the factor V Leiden mutation [19].

Given the interaction of DIM in the estrogen- and testosterone-related pathways, the limited studies of DIM have focused on breast, cervical, and prostate cancer research. Malignancies are a risk factor for thromboembolism, and any additional risk that DIM confers is unclear. Further research into the beneficial and adverse effects of DIM is necessary. Such research may involve the dose-dependent effect of DIM. DIM is ingested naturally in the diet, but supraphysiological dosing may promote the development of VTE.

This single case of a patient developing acute DVT and PE while taking DIM with a long-term complication of chronic DVT reiterates the importance of obtaining information about supplements. In the appropriate clinical context, such as the presence of risks for VTE, clinicians need to have a low threshold for further evaluation for VTE. Other cases of VTE in the setting of DIM should be reported.

## Figures and Tables

**Table 1:** Timeline of events.

Time	Event
10 years ago	The patient had a possible pulmonary embolism.

6 years ago	The patient ceased smoking tobacco.

1 year ago	The patient resumed smoking tobacco. PSA was less than 3 ng/mL.

13 to 19 months ago	The patient used over-the-counter testosterone supplements.

4 months ago	Colonoscopy found a benign polyp.

3 to 4 months ago	The patient started using over-the-counter diindolylmethane.

5 weeks ago	The patient presented to the Emergency Department and was thought to have right sciatica.

3 weeks ago	The patient presented to the Emergency Department and was thought to have costochondritis.

Day 0	A lower extremity venous Doppler study found deep venous thromboembolism in his right lower extremity. Computed tomography of the chest found bilateral pulmonary embolism with probable right middle lobe infarction. The patient was hospitalized and started on anticoagulation.

5 months later	A lower extremity venous Doppler study found chronic deep venous thromboembolism in his right lower extremity.
